# Managing gender-based violence patients: implications for the practices and attitudes of emergency medical care providers

**DOI:** 10.11604/pamj.2024.49.108.38454

**Published:** 2024-12-04

**Authors:** Fose John Mohokare, Nokuthula Tlalajoe-Mokhatla

**Affiliations:** 1 Department of Health Free State, Free State College of Emergency Care, Free State, South Africa,; 2 Faculty of Health Science, University of the Free State, Free State, South Africa

**Keywords:** Attitudes, beliefs, emergency medical care providers, emergency medical service, gender-based violence (GBV), GBV patients

## Abstract

**Introduction:**

Gender-Based Violence (GBV) is one of the most widespread human rights abuses in the world. Although women and girls are often the targets of GBV, men and boys can also suffer from it. Therefore, this study investigated the practices and attitudes of Emergency Medical Care Providers (EMCPs) in managing GBV patients.

**Methods:**

this study applied self-administered questionnaires and analyzed them quantitatively. The information was gathered from 130 qualified and practicing EMCPs in Bloemfontein working within the public sector of Emergency Medical Service (EMS).

**Results:**

the results showed that a significant association was observed among socio-demographic characteristics such as gender, educational status, marital status, and how EMCPs managed GBV cases. On the other hand, a non-significant association was observed among age, year of experience, and how EMCPs managed GBV cases. Furthermore, a significant association was observed among the beliefs of the society, attitudes of the environment towards abuse victims, and exaggeration of suspected GBV patients influences the management of GBV patients.

**Conclusion:**

the study concluded that while the current EMCPs in Bloemfontein, Free State South Africa, are relatively educated, there is still room for improvement to cover the growing demands of the job to be done for GBV cases.

## Introduction

Gender-based violence (GBV) occurs as a cause and consequence of gender inequities. It includes a range of violent acts (i.e. sexual GBV, domestic violence, intimate partner violence, violence against women, sexual violence, sexual exploitation, sexual abuse, and sexual harassment) predominantly committed by males against females, within the context of women and girls subordinate status in society, and often serves to retain this unequal balance [1]. This does not mean that all acts against a woman are gender-based violence, or that all victims of gender-based violence are female. The surrounding circumstances where men are victims of sexual violence could be a man being harassed, beaten, or killed because they do not conform to the view of masculinity, which is accepted by society [2]. Thus, when speaking about the individual involved in a GBV incident, it is important to use clear terminology. Depending on the context of the discussion, the words used to refer to the person affected may change. Emergency Medical Care Providers (EMCPs) may refer to the individual as a “patient”. Law enforcement officials may refer to the person as a “victim”. In a court of law, the person may be called the “complainant”. That same person may also be referred to as a survivor when being discussed within the realm of advocacy. Each term has nuances and connotations. For clarity and consistency, and coming from a medical perspective, this paper will refer to these individuals as “patients”. Gender-based violence (GBV) continues to be one of the most widespread human rights abuses in the world, and it is a complex problem facing the healthcare and public health systems [3]. Gender-based violence is a broad concept that can be categorized as rape, human trafficking, forced prostitution, and domestic violence, which is the most egregious manifestation of gender inequality by keeping women and girls subordinate and under the control of men [4]. Koenig *et al*. mentioned that sexual assaults can affect people of any age, sexual identity, or gender, with lasting effects on patients and their families. Women and girls are often the targets of GBV, and while no woman is immune from it, not all women, are, however, equally at risk [2]. The key risk factor for experiencing GBV is being female due to factors such as age, financial dependence, poverty, disability, homelessness, and insecure immigration status can heighten women´s vulnerability to abuse or entrap them further in it [5]. Gupta *et al*. used a cross-sectional observational study done in the Department of General Practice and Emergency Medicine of BP Koirala Institute of Health Sciences from January 2014 to December 2014. They discovered that domestic violence is a particularly insidious form of gender-based violence where 71.9% of the total number of patients enrolled were female [6]. In the place where they should feel the greatest safety and security, the family, women often face terror in the form of physical, psychological, sexual, and economic abuse. Highlighting how females are the most common victims. Contrarily, GBV takes place worldwide, irrespective of age, sex, religion, class or caste. The shocking truth is that violence against women and girls takes place in all countries, in homes, workplaces, schools, and communities [7]. Young women are at high risk of all forms of abuse, yet often this can be overlooked or minimized, particularly in their teenage years. Meanwhile, older women´s experiences may be invisible or misunderstood as elder abuse [8]. However, men and boys can also suffer from it, especially if they are perceived to be acting outside the prescribed social norms [2,9]. Factors such as GBV can be contextualised in a domestic context (within a past or present relationship) and are generally or commonly perpetrated by a male partner. This is unfortunately experienced by the women as harmful and destructive to themselves physically, emotionally, socially, and psychologically [10]. Harland *et al*. [11] study was to estimate the prevalence of intimate partner violence in an emergency department by sexual orientation and gender identification using a cross-sectional survey of adult patients (n=1,136) collected for one year. Prevalence of intimate partner violence was significantly higher in lesbian, gay, bisexual, transgender, and questioning (LGBTQ) patients than in heterosexuals, prevalence was highest among bisexuals and gay men. Intimate partner violence did not differ significantly in females versus males. Moreover, gay patients and females had significantly higher odds of reporting physical or sexual intimate partner violence than heterosexuals and males. This study is among the first to report intimate partner violence prevalence by sexual orientation in an emergency department patient population. The reported intimate partner violence was higher among LGBTQ patients than heterosexual patients. It is perpetuated by a behavioural pathology of the perpetrator and victim that may include escalating violence, concealment, shame, low self-esteem, risk-taking, alcohol and substance abuse in a context of power and control based on one´s gender [12]. It is a population-level issue that affects everyone, while also heavily concentrating among specific vulnerable populations [3]. Unfortunately, the ongoing coronavirus disease 2019 (COVID-19) pandemic has increased the frequency of domestic violence [2].

Sharma *et al*. [13] study highlighted that GBV cases should be managed by means of protocols and captured as a data set for quality monitoring purposes or sentinel surveillance. Emergency Medical Care Providers should have a clear understanding of how to approach suspected GBV patients. They should start with screening, provide supportive care, assess the risk as well and be able to provide relevant information to the victim on how to get a protection order [14]. However, for the most part, the activities of EMS in relation to GBV patients are still scarce. This is largely due to the fact EMS in South Africa for GBV patients is still in the developmental stages and the proper coordination needed in this critical area is still largely deficient. As first responders at the scene of domestic violence calls where personal injuries have occurred, it is possible that EMCPs routinely identify, report, and assist patients of domestic violence. Emergency Medical Care Providers are, therefore, uniquely positioned to help abused women by both treating the injuries they may have sustained and providing them with the necessary support, resources, and information; or when that is not possible, by alerting the receiving hospital about the patient´s condition before parting the scene. Early identification, comprehensive management, documentation of the abuse and injuries sustained, and appropriate referral, may be among the most effective strategies to prevent further injury and stem the medical and psychological consequences of domestic violence [15]. To the authors´ best knowledge, none of the studies assessed the implications of practices and attitudes of emergency care providers managing suspected GBV patients. Previous studies mentioned above focused on the impact of using an emergency response infrastructure; screening for domestic violence; general practice and emergency medicine and estimating the prevalence of intimate partner violence. Therefore, this study responds to the issue of paucity of literature directed towards EMCPs that addressed the management of patients who are suspected GBV patients. This is because the requisite technical training needed for their job delivery is fundamental to their success as professionals, thus, this is the research gap that this study filled.

## Methods

**Study design:** a descriptive and quantitative method was adopted to describe the practices and attitudes of EMCPs in the approach and treatment of GBV patients in Bloemfontein, Free State, South Africa.

**Study setting:** we conducted the study in all provincial EMS institutions in Bloemfontein.

**Study participants and sampling:** all qualifying and practicing EMCPs in Bloemfontein working within the public sector of Emergency Medical Service (EMS), who often provided emergency medical care to patients of GBV. The researchers ensured that all participants have one of the following qualifications in Emergency medical care (EMC), Basic ambulance assistant (BAA), Ambulance Emergency Assistant (AEA), Emergency Care Technician (ECT), B-Tech: emergency medical care, Bachelor of Health Science in Emergency Medical Care, Emergency care assistance (ECA) and Diploma in Emergency Medical Care (DEMC).

**Data collection:** 130 self-administered questionnaires were hand-delivered to all the different Bloemfontein provincial EMS institutions. The participants were informed that they had a minimum of two days to complete the questionnaires and to drop it into a closed marked box placed in a secure area in the institution.

**Data management and analysis:** the quantitative analytical method used for this work allowed for the data collection on the specific location of the study, the emotional capacity of the participants, and their readiness for the study.

**Ethical considerations:** the study received ethics approval from the University of the Free State Health Sciences Research Ethics Committee (UFS-HSD2020/1769/2809). Permission to carry out the project was also obtained from all provisional EMS institutions. All study participants provided written informed consent prior to administration of the questionnaire. The questionnaires could not be matched with any participant, since no names or personal identifiers appeared on the questionnaire. Each questionnaire was identified using a unique bar code, which ensured the participants´ anonymity.

## Results

**Socioeconomic characteristics:** as shown in Table 1, the demographic data of the 130 participants who were recruited shows; that 0.8% were within 20 - 25 years; 18.5% fell within 25 - 35 years; 57.7% were within 35 - 45 years; and 23.1% were either 45 years old and even above. The participants consisted of 84 females and 46 males (64.6% and 35.4%, respectively). Similarly, the analyzed data of the educational statuses of the study participants showed that 48 participants (36.9%) completed high school, 76 participants (58.5%) completed their colleges, and 4 study participants (3.1%) completed university degrees, while only two (2) participants (1.5%) completed their postgraduate degrees. Furthermore, 23.8% had 1 - 5 years of work experience, 23.8% already had 5 -10 years of work experience, 37.7% already put in 10-15 years and 14.6% have worked for at least 15 years and above as EMCPs.

**Table 1:** socio-economic characteristics of participants

Variable	Categories	Frequency n=130	Percent (%)
Age (years)	20-25	1	0.8
	25-35	24	18.5
	34-45	75	57.7
	45 and above	30	23.1
Gender	Male	46	35.4
	Female	84	64.6
Educational status	High school	48	36.9
	College	76	58.5
	Degree	4	3.1
	Postgraduate	2	1.5
Years of experience	1-5 years	31	23.8
	5-10 years	49	37.7
	15 years and above	19	14.6

### Practices and attitudes of emergency medical care providers in the management

**Suspected gender-based violence patients:** Table 2 shows a significance of 71.5% admitting that they do not often receive enough information about the management of GBV patients; while another significant 56.9% said that they often came across physical abuse as the predominant form of abuse. Similarly, 22.3% confessed that they have had encounters with patients of sexual abuse, 14.6% admitted that they have had to attend and cater to patients and survivors of emotional abuse, while 1.5% mentioned that they have had encounters with patients of both spiritual and economic abuse. Furthermore, a significant 83.8% agreed that both genders suffer from GBV, while 92.3% agreed that minors also suffer from GBV and that all genders must be managed equally when abused. The majority of participants 95.4% agreed that the psychological status of an abused patient must be considered when managing a suspected GBV patient. Additionally, 97.7% mentioned that suspected abuse patients must have sessions with counselors. A significant majority of 80.8% further admitted that gender preferences should not be considered as a factor when managing suspected GBV cases. Additionally, 88.5% said that spiritual abuse should also be managed as well when managing suspected GBV patients, while virtually all study participants also agreed that suspected GBV patients should be referred to experts for proper management.

**Table 2:** practices of emergency medical care providers in managing suspected gender-based violence patients

Variable	Categories	Frequency n=130	Percent (%)
Do you think you get enough information about the management of GBV patients?	Yes	37	28.5
	No	93	71.5
What type of GBV cases do you often come across in the course of your practice? **n=126**	Physical abuse	74	56.9
	Sexual abuse	29	22.3
	Emotional abuse	19	14.6
	Spiritual abuse	2	1.5
	Economic abuse	2	1.5
Do you think that it is only women that are affected by GBV?	Yes	26	20.0
	No	104	80.0
Do you think that both genders suffer from GBV?	Yes	109	83.8
	No	21	16.2
Do you think that minors also suffer from GBV?	Yes	120	92.3
	No	10	7.7
Do you think that all genders must be managed equally when abused? **n=128**	Yes	120	92.3
	No	8	6.2
Do you think that psychological status of an abused patient must be considered when managing a suspected GBV patient? **n=128**	Yes	124	95.4
	No	4	3.1
Do you think that suspected abused patients must have sessions with counsellors? **n=128**	Yes	127	97.7
	No	1	0.8
Do you think treatment is necessary when abuse is not physical? **n=127**	Yes	102	78.5
	No	25	19.2
Do you think one gender is more important than the other when managing suspected GBV patients? **n=128**	Yes	23	17.7
	No	105	80.8
Do you think that spiritual abuse must be managed as well when suspected in GBV patients? **n=126**	Yes	115	88.5
	No	11	8.5
Do you think that suspected GBV patients should be referred to experts for proper management? **n=128**	Yes	124	95.4
	No	4	3.1

**Knowledge of emergency care:** the frequency distribution of the knowledge of emergency care given to suspected GBV patients. 35.5% of participants had high knowledge of the care administered to patients of GBV, while a significant 64.6% admitted that they had moderate knowledge of the emergency medical care for suspected GBV patients.

**Attitudes of emergency medical care providers:** Table 3 helps to understand the attitudes of the EMCPs while approaching and treating suspected patients of GBV. From the results, 27.7% admitted that they received little professional training concerning the management of GBV cases, especially as it affects their practice, while only 13.8% disagreed with that assertion. Furthermore, 48.5% disagreed with the popular opinion that GBV is a social problem, and it is not relevant to their daily practices. Similarly, 32.3% admitted that the beliefs of the society where they live affected their practice in managing suspected GBV patients, while 43.8% agreed that the attitudes towards patients of violence in their lived environment affected their practice in managing suspected GBV patients. Also, 30% mentioned that the perceived threats to their personal safety are among the factors that affected their attitude toward the management of suspected GBV patients. However, 10.8% opined that their attitudes towards suspected GBV patients have never been shaped by their environment or any safety concerns.

**Table 3:** attitudes of emergency medical care providers towards suspected gender-based violence patients

Variables	Strongly agree- N (%)	Agree- N (%)	Undecided- N (%)	Disagree- N (%)	Strongly disagree- N (%)
I received little professional training on how to manage cases of GBV and this affected my practice in managing suspected GBV patients	36 (27.7%)	35 (26.9%)	4 (3.1%)	35 (26.9%)	18 (13.8%)
GBV is a social problem that is not relevant to my daily practices	7 (5.4%)	22 (16.9%)	1 (0.8%)	63 (48.5%)	33 (25.4%)
The beliefs of the society that I live affect my practice in managing suspected GBV patients	33 (25.4%)	42 (32.3%)	10 (7.7%)	29 (22.3%)	16 (12.3%)
The attitudes towards victims of violence in my environment affect my practice in managing suspected GBV patients	19 (14.6%)	57 (43.8%)	14 (10.8%)	19 (14.6%)	17 (13.1%)
The threats to my personal safety is part of the factors that affect my attitude towards the management of suspected GBV patients	25 (19.2%)	39 (30%)	17 (13.1%)	29 (22.3%)	14 (10.8%)
I do not think that attention must be paid to the emotional state of the suspected GBV patients	6 (4.6%)	12 (9.2%)	7 (5.4%)	0 (0.0%)	45 (34.6%)
I believe that the suspected GBV patients are the cause of their abuse	12 (9.2%)	21 (16.2%)	10 (7.7%)	45 (34.6%)	42 (32.3%)
Exaggeration of suspected GBV patients about abuse affects my practice in managing GBV patients	15 (11.5%)	47 (36.2%)	20 (15.4%)	35 (26.9%)	11 (8.5%)
The policy and legal issues of my environment affect my practice in managing suspected GBV patients	14 (10.8%)	34 (26.4%)	25 (19.2%)	42 (32.3%)	13 (10.0%)
The little understanding that I have about GBV affects my practice which makes me underestimate the future injuries of suspected GBV patients, while managing them	25 (19.2%)	40 (30.8%)	23 (17.7%)	25 (19.2%)	17 (13.1%)

**Figure 1:**
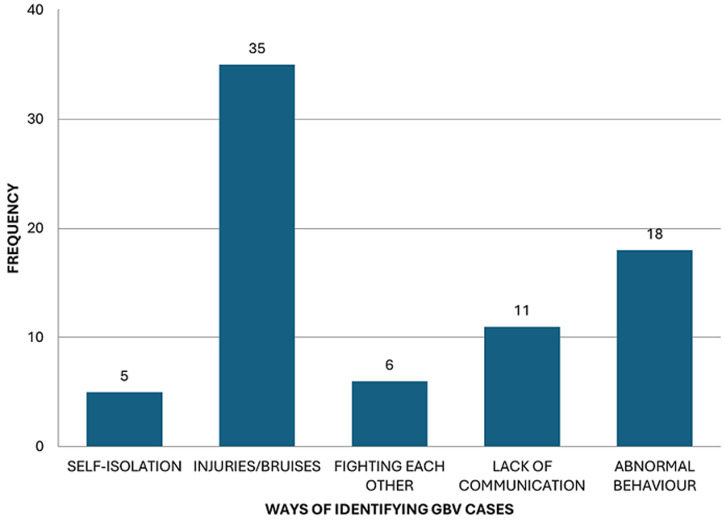
frequency distribution of how emergency medical care providers identified gender-based violence cases

**How emergency medical care providers identify gender-based violence cases:** subsequently, from Figure 1, EMCPs admitted that they were able to identify suspected GBV cases through the following mechanisms: 13.8% said they identified them through the abnormal behaviour displayed by the patients, 8.5% admitted that they identified them through lack of proper communication by the patients, while 4.6% mentioned that they identified them through incessant fighting of the partners.

**Figure 2:**
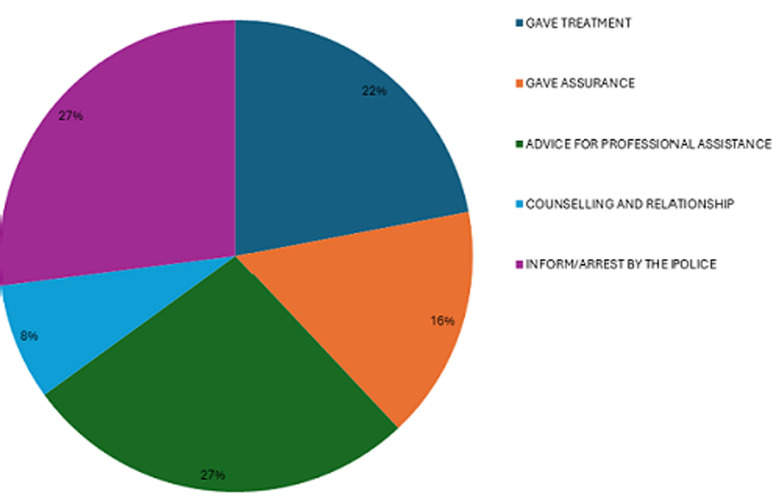
how emergency medical care providers managed emergency gender-based violence cases differently from other cases

**How did you manage gender-based violence cases:** furthermore, from Figure 2, 16% mentioned that they recommended that the patients of GBV undergo self-isolation as assurance, to prevent their abusers from having access to them, 27% mentioned that they offered professional assistance to the patients, and 8% admitted that they provided thorough professional counseling to them. Similarly, 27% said that they had to involve the police in the matter, while 22% mentioned that they administered prompt treatment to the suspected patients.

**Figure 3:**
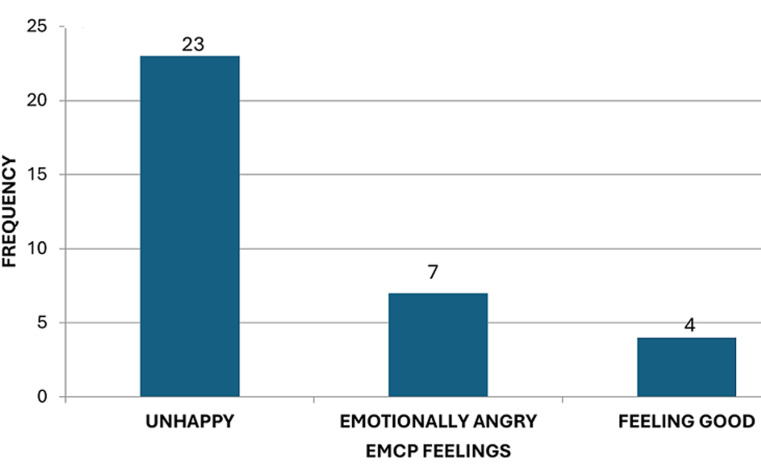
emergency medical care providers´ feelings about gender-based violence cases

**Emergency medical care providers feelings about gender-based violence cases:** as seen in Figure 3, the frequency distribution of the EMCPs experiences and feelings about suspected cases of GBV are spread as follows; 22.5% were unhappy about the situation of their patients, 6.9% were emotionally angry, while 3.8% felt good about the situation.

## Discussion

The study found that there are more people who can be described as being in their mid-life (35- 45 years) in the profession and this may be because the EMCPs should be those who have acquired a reasonable level of life experiences. These findings are consistent with the findings by [16], which suggest that EMCPs within the Free State province are relatively young (median age group 36-40 years). The female-to-male ratio was contrary to the traditional findings where the EMC sector is considered a male-dominated field, as well as the findings suggesting that the EMC profession is still dominated by male practitioners in the Free State province [17]. However, this finding is in agreement with the conclusions of the United Nations Special Rapporteur for Africa (2011) report, where the study reported that there are often more female EMCPs as professionals when compared with male EMCPs. The analyzed data on the educational statuses of the study participants is in agreement with what was stated by Mothibi *et al*. [17], the majority of the EMCPs in the Free State province are in possession of qualifications obtained from 4 - 6 weeks to 4 months, while minority are among those who completed a university degree. From the preceding results, it is obvious that the majority of the participants (37.7%) were those who fell within the 10-15 working years experience bracket.

Participants of this study underlined that they lacked information about the management of GBV patients and that in their experiences it is only a handful of GBV patients encountered both spiritual and economic abuse. These findings are in conflict with existing literature by the World Health Organization, where the study concluded that the third category of GBV that women face globally is economic abuse, only preceded by sexual and physical abuse [7]. In relation to both genders suffering from GBV, Harland *et al*. opine that, LGBTQ subjects had higher odds of any GBV than heterosexuals while gay subjects had significantly higher odds of physical or sexual GBV than heterosexuals [11]. However, in practice, all genders can be abused or suffer from GBV, it then becomes imperative for practitioners of emergency care to treat all patients equally without recourse to their gender or sexual orientation. These findings complement the discoveries reported by Aschman *et al*. [18] stating that there is a need to look beyond just screening for injuries among GBV patients. Initiatives such as, at the very least, making patients feel that someone cares about their situation and gaining access to psychosocial services and potential coping strategies, and, at best, reducing experiences of violence and the termination of the abusive relationship, must also come into consideration when attending to GBV patients. The comforting aspect is that EMCPs in Bloemfontein have demonstrated to have qualified knowledge of the care administered to suspected patients of GBV, but there is still much room for improvement. Subsequently, these improvements should be all-encompassing and touch all needed areas. Moreover, these findings also reflect the historical education and training of EMC in South Africa which was implemented using short vocational courses [16].

In addition to the above suggestion of improving the EMCPs service delivery to suspected GBV patients. Table 3, further highlights a lot of social awareness that must be done to sensitize the public about the menace of GBV that has been described GBV as a global social and public health problem [3]. Moreover, the beliefs of the society have an impact in managing suspected GBV patients, because it was reported that several factors may contribute to the geographical concentration of GBV cases in specific neighborhoods. Alcohol and substance use are often associated with increased rates of harm and violence, and areas with a concentration of bars and clubs have been documented as risk factors for aggression and violence [3]. Personal safety is patently a call for concern among EMCPs and this has a direct effect on their attitudes towards the management of suspected GBV patients. Two-thirds of EMS workers have been attacked on the job, meaning that the same individuals who are called out to help GBV patients are at high risk of being patients of violence and abuse. With the increasing number of attacks on EMCPs. Several EMCPs who are working in a pre-hospital environment are exposed to hostile working conditions and this may affect how they approach patients in general [19]. Workplace violence experienced by EMS has been linked to psychological injuries in the form of stress, anxiety, and Post-traumatic stress disorder (PTSD) [20].

Even though the EMCPs in Bloemfontein had demonstrated to have qualified knowledge of the care administered to suspected patients of GBV, they did however admit that they lack training on how to approach or treat suspected GBV patients, with 65% of EMCPs noting that they only have moderate knowledge of emergency care for GBV patients, while a small percentage (35%) reported they have high knowledge. Naidoo [21], places emphasis on training and routine screening of calls as an important factor in assisting EMCPs to better understand how to deal with GBV patients. Identification of suspected GBV was done through a few mechanisms (Figure 1). Butler [22], thus concluded that if EMCPs are very patient and attentive enough to the symptoms of GBV patients, they can easily know that they are suffering. However, one can argue and suggest that perhaps formal training should first be put into place before having such expectations from the EMCPs. Also, support structures that equip EMCPs to address their concerns such as personal safety while attending to suspected GBV patients must also be accessible. Because it is evident from Figures 2 and 3 that there are a reasonable number of emergency care providers who feel unhappy and angry about the prevalence of GBV in Bloemfontein, there are still some who are yet to understand the magnitude and gravity of GBV. However, they too do not consider themselves fully fit to function effectively in meeting the requirements of successfully managing suspected GBV patients through appropriate approaches and treatment knowledge, resulting from the lack of formal training and education.

## Conclusion

This study demonstrated the effects that practice, and attitude has towards managing suspected GBV patients. With that said, a few aspects are said to be considered in aiding to bridge the gap between the existing skills and knowledge of managing suspected GBV. Such aspects range from the socio-demographic characteristics such as gender, educational status and marital status in how EMCP identify GBV cases. Moreover, the study is also proposing that the beliefs of the society, attitudes about the environment towards abuse victims and the exaggeration of suspected GBV patients also be considered as this influences the management of GBV patients.

### What is known about this topic

- The focus was mostly on the impact of using an emergency response infrastructure; screening for domestic violence; general practice and emergency medicine and estimate the prevalence of intimate partner violence;
- Frontline workers such as emergency medical care providers play a vital role when it comes to dealing with gender-based violence.

### What this study adds

- Implication of practices and attitudes of emergency medical care providers managing suspected gender-based violence patients;
- Communicating aspects that need to be considered in closing the gap of effectively managing suspected gender-based violence patients;
- Highlighting requisite technical training needed for their job delivery among gender-based violence handling suspected gender-based violence cases.

## References

[1] Willman AM, Corman C (2013). Sexual and gender-based violence: what is the World Bank doing, and what have we learned? A strategic review. Social Development.

[2] Koenig K, Benjamin SB, Bey CK, Dickinson S, Shores M Violence: Recognition, Management and Prevention. The Journal of Emergency Medicine. 2020;.

[3] Muldoon K, Galway L, Reeves A, Heimerl M, Sampsel K (2021). Geographies of Sexual Assault: A Spatial Analyses to identify Neighbourhoods Affected by Sexual and Gender-Based Violence. Journal of Interpersonal Violence.

[4] Abdelnour S, Poon M (2013). Is Sexual Violence being Adequately Addressed in Global Conflict Zones. Risk and Regulation.

[5] Sultana A (2010). Patriarchy and women s subordination: a theoretical analysis. Arts faculty journal.

[6] Gupta P, Bhandari R, Khanal V, Gupta S (2018). A cross-sectional study on domestic violence in emergency department of Eastern Nepal. J Family Med Prim Care.

[7] World Health Organization (2013). Responding to Intimate Partner Violence and Sexual Violence against Women: WHO Clinical and Policy Guidelines.

[8] McCloskey KA, Raphael DN (2005). Adult perpetrator gender asymmetries in child sexual assault victim selection: results from the 2000 National Incident-Based Report System. J Child Sex Abus.

[9] World Health Organization Violence and Health in the WHO Africa Region.

[10] Matheson FI, Daoud N, Hamilton-Wright S, Borenstein H, Pedersen C, O'Campo P (2015). Where did she go? The transformation of self-esteem self-identity and mental well-being among women who have experienced intimate partner violence. Womens Health Issues.

[11] Harland K, Peek-Asa C, Saftlas AF (2021). Intimate Partner Violence and Controlling Behaviors Experienced by Emergency Department Patients: Differences by Sexual Orientation and Gender Identification. J Interpers Violence.

[12] World Health Organization (2011). Global and Regional Estimates of Violence against Women: Prevalence and Health Effects of Intimate Partner Violence and Non-Partner Sexual Violence.

[13] Sharma V, Ausubel E, Heckman C, Rastogi S, Kelly JTD (2022). Promising practices for the monitoring and evaluation of gender-based violence risk mitigation interventions in humanitarian response: a multi-methods study. Confl Health.

[14] Health Professions Council of South Africa (2016). Guidelines for good practice in the health care professions. Ethical guidelines for good practice with regard to HIV.

[15] Artz L, Meer T, Aschman G (2018). Legal duties, professional obligations or notional guidelines? Screening treatment and referral of domestic violence cases in primary health care settings in South Africa. Afr J Prim Health Care Fam Med.

[16] Rowland M, Adefuye A (2022). Human errors and factors that influence patient safety in the pre-hospital emergency care setting: perspectives of South African emergency care practitioners. Health SA.

[17] Mothibi J, Jama M, Adefuye A (2019). Assessing the knowledge of emergency medical care practitioners in the Free State, South Africa, on aspects of pre-hospital management of psychiatric emergencies. PanAfr ican Medical Journal.

[18] Aschman G, Meer T, Artz L (2012). Behind the screens: domestic violence and health care practices. Agenda.

[19] Lambert V, Westwood R (2019). Students´ views on the need for hostile environment awareness training for South African emergency medical care students. Afr J Health Prof Educ.

[20] Relias Learning Two-Thirds of EMS Workers Have Been Attacked on Job Hospital Employee Health Atlanta.

[21] Naidoo N (2017). Gender-based violence: strengthening the role and scope of prehospital emergency care by promoting theory, policy and clinical praxis.

[22] Butler MW (2011-2013). Experiences of Free State Emergency Medical Care Practitioners Regarding Pediatric Pre-Hospital Care (Doctoral Dissertation, University of the Free State). Challenging-GBV-Worldwide Cares Program Experience.

